# Development of a prediction model for urinary tract infection risk after open reimplantation in children with primary unilateral vesicoureteral reflux: A multicentre study

**DOI:** 10.1002/bco2.70111

**Published:** 2025-11-17

**Authors:** Khadija Ismail, Mohamad Moussa, Bilal Aoun, Mohamad Abou Chakra, Anthony Kallas‐Chemaly, Priyank Yadav, Christian Kruppa, Katrin Schuchardt, Alexandra Wilke, Pascale Salameh, Amal Al‐Hajje

**Affiliations:** ^1^ Lebanese University, Doctoral School of Sciences and Technology Beirut Lebanon; ^2^ Clinical and Epidemiological Research Laboratory, Faculty of Pharmacy Lebanese University Beirut Lebanon; ^3^ Urology Department, Zahraa Hospital, University Medical Centre Lebanese University Beirut Lebanon; ^4^ Faculty of Medical Sciences, Urology Department Lebanese University Beirut Lebanon; ^5^ Division of Paediatric Nephrology American University of Beirut‐Medical Centre Beirut Lebanon; ^6^ Department of Urology University of Iowa Iowa City IA USA; ^7^ Hôtel‐Dieu de France University Hospital, Paediatric Urology Saint Joseph University Beirut Lebanon; ^8^ Mount Lebanon University Hospital, Paediatric Urology Balamand University Beirut Lebanon; ^9^ Department of Urology and Renal Transplantation Sanjay Gandhi Postgraduate Institute of Medical Sciences Lucknow Uttar Pradesh India; ^10^ Department of Paediatric Surgery University Hospital Dresden, Technical University Dresden Dresden Germany; ^11^ INSPECT‐LB (Institut National de Santé Publique, d'Epidémiologie Clinique et de Toxicologie‐Liban) Beirut Lebanon

**Keywords:** children, nomogram, risk assessment, urinary tract infection, vesicoureteral reflux

## Abstract

**Objectives:**

To develop a predictive model for symptomatic postoperative febrile urinary tract infections (UTIs) in children undergoing open reimplantation for vesicoureteral reflux (VUR) and evaluate the association with VUR recurrence.

**Patients and methods:**

This multicentre retrospective study included children with unilateral VUR (grades III–V) who underwent open Cohen or Lich‐Gregoir reimplantation (2010–2022), had recurrent febrile UTIs, and ≥1 year follow‐up. Analyses used 10‐pooled multiple imputation, with complete case for sensitivity. Full and Least Absolute Shrinkage and Selection Operator (LASSO) Weibull regression models with centre clustering, bootstrapping, and 10‐fold cross‐validation identified predictors. Prediction used demographic, clinical, procedural and antibiotic factors. The non‐scaled LASSO model informed the nomogram, evaluated using C‐indices, calibration and decision curve analysis (DCA). UTI and VUR recurrence were analysed via cumulative incidence.

**Results:**

A total of 404 children (median age 8 (6–9) years; follow‐up 2.3 (2.0–3.3) years, 233 complete‐case) were analysed. Median preoperative febrile UTIs were four, 74.5% had antibiotic resistance and median postoperative prophylaxis was two days. The 3‐year cumulative incidence of postoperative UTI was 27.2% (95% CI: 22.9–31.6). LASSO‐significant predictors included operative time (HR 1.10, 95% CI 1.03–1.16); in sensitivity analyses, prior injection (HR 2.08, 95% CI 1.88–2.30) and postoperative antibiotic duration (HR 0.81, 95% CI 0.69–0.97) were also significant. The nomogram included preoperative fever, antibiogram resistance, renal defect, VUR phase, prior injection, surgical indication, catheterization, hospitalization and stenting. The model performed well (C‐indices = 0.743; calibration slope = 1), with DCA supporting clinical utility for 10–40% predicted risk. Recurrent VUR grade ≥II after 12 months (3.3%–12.7% at 1–3 years, n = 273) did not increase UTI risk.

**Conclusions:**

Children with unilateral dilating VUR remained at risk of postoperative febrile UTIs. The nomogram can assist in identifying high‐risk children for targeted interventions, but requires external validation and refinement.

## 1 INTRODUCTION

Vesicoureteral reflux (VUR) is a common congenital anomaly characterized by the backward flow of urine from the bladder into the ureters.^1^ It increases the risk of recurrent febrile urinary tract infections (UTIs), which may cause renal scarring, a major concern in children.^2^ Among children with febrile UTIs, renal scarring has been reported in up to 54% of those with VUR, compared to 12% in those without VUR.^3^, ^4^ Effective management of VUR is therefore considered important for reducing the risk of kidney damage.

Ureteral reimplantation is the standard surgical treatment for dilating VUR, aiming to correct the reflux, prevent febrile UTIs and preserve renal function.^1^ The resolution of VUR is currently investigated only following febrile UTI episodes.^1^, ^5^ In this context, postoperative UTI recurrences have been reported in a limited number of studies, with rates ranging from 0% to 27%, and in some cases reaching up to 40%.^6^, ^7^, ^8^, ^9^, ^10^, ^11^, ^12^, ^13^, ^14^, ^15^, ^16^, ^17^ Risk factors for recurrence remain poorly defined, with only one prior study identifying sex, preoperative breakthrough infections, operative success and voiding dysfunction as potential contributors.^12^

VUR resolution is assessed using voiding cystourethrography (VCUG), an invasive imaging modality that may cause significant discomfort.^18^ In light of this, predicting the long‐term risk of postoperative UTIs may help guide risk stratification, optimize follow‐up strategies and inform management decisions. Although several predictive models have been developed to estimate UTI recurrence in children treated with or without antibiotic prophylaxis,^2^, ^19^, ^20^ no model has specifically addressed the risk of UTI following ureteral reimplantation.

This multicentre study aimed to estimate the long‐term recurrence of UTIs following open Cohen or Lich‐Gregoir ureteral reimplantation. It analysed the influence of demographic, clinical, procedural and antibiotic‐related factors on postoperative UTI risk. The study developed a novel nomogram to predict recurrence and decision curve analyses to evaluate the clinical utility of 1‐ and 3‐year predictions. In addition, it assessed the rates of VUR recurrence and their association with postoperative UTIs.

## 2 PATIENTS AND METHODS

### 2.1 Study design and population

This multicentre, retrospective cohort study involved five tertiary medical centres in Asia and Europe. We excluded one centre due to its lack of eligible surgical VUR patients, which reflects its role as a referral centre or its more conservative approach. Institutional Review Board approvals were obtained from the Lebanese centres, with verbal data‐sharing agreements from India and Germany. Data collection was carried out by local physicians using a standardized form.

Children (> 1 year old) diagnosed with primary unilateral dilating VUR grade III–V and treated surgically between June 2010 and September 2022, with either the Cohen cross‐trigonal or Lich‐Gregoir open ureteral reimplantation techniques, were included. Eligibility also required at least one culture‐proven febrile UTI (>10^5^ colony‐forming units/mL) and a minimum of one year of follow‐up. Surgeries were performed for several clinical indications, including symptomatic high‐grade VUR, defined as reflux associated with recurrent UTIs, renal damage, hydronephrosis or pain. Excluded children were those with secondary VUR, repeated or tapering reimplantation, bladder and bowel dysfunction, posterior urethral valves, previous bladder surgery or reflux‐related anomalies (ureterocoeles, duplex systems, megaureter or ectopic ureter).

Children were characterized based on available medical records, including voiding cystourethrograms (VCUG), renal ultrasounds, dimercaptosuccinic acid (DMSA) scans, lab results, operative notes and physical exams. Monthly visits during the first year were part of the routine follow‐up protocols, including physical examinations, urinalyses and renal ultrasounds. Thereafter, annual evaluations were conducted, consisting of blood pressure measurement, urinalysis and imaging studies (ultrasound, VCUG or DMSA) as clinically indicated. Any frequent follow‐up during the first year was unlikely to introduce bias, as all postoperative UTIs were clinically diagnosed based on the presence of symptoms. Short‐term antibiotic prophylaxis (1 day–3 months) was administered based on the patient's history, local resistance and institutional protocols, and included in the analysis to account for its potential residual effect on long‐term postoperative UTIs. Antibiotics used were nitrofurantoin (2 mg/kg) until stent removal or trimethoprim‐sulphamethoxazole (TMP‐SMX) for up to three months, with choice and duration individualized. The study followed TRIPOD‐Cluster 2023 reporting guidelines.^21^

### 2.2 Outcomes

The primary outcome was postoperative febrile UTIs, modelled as the time to the first febrile UTI occurring more than one month after surgery. Fever (≥38°C), urinary symptoms and a culture‐proven infection (>10^5^ CFU/ml) with pyuria (>10 leukocytes/mL) were the criteria used to define this outcome.^1^, ^5^ Urine samples were collected if symptoms suggested a UTI, using age‐appropriate methods. For children who were toilet‐trained, midstream clean‐catch urine samples were obtained through volitional spontaneous urination, while for children who were not, sterile catheterization was used. These methods ensured reliable urine collection tailored to developmental stages and clinical standards. The secondary outcome was VUR recurrence, defined as VUR grade ≥ II in the reimplanted ureter, identified either on the initial 12‐month postoperative cystogram or on repeated VCUGs following a febrile UTI. This definition did not include grade I VUR, as it is often considered clinically insignificant and may resolve spontaneously without further intervention.^22^

### 2.3 Predictors

The predictive model used a limited set of well‐defined predictors, including demographic, clinical, procedural and antibiotic‐related factors. Predictor selection was motivated by prior models for VUR resolution, preoperative UTI risk and expert input in paediatric urology.^2^, ^20^, ^23^, ^24^, ^25^

Key factors included age, body mass index (BMI), dilating VUR grades (III–V) as per the International Reflux Study scale, and ureteral dilation indexed to L1–L3 distance (≥ 7 mm).^24^ Acute pyelonephritis (APN) and renal scarring on the affected side were assessed via ultrasound and confirmed by DMSA scans, identifying scarring by reduced uptake and contour loss.^3^ Surgeon experience was categorized by annual case volume (<50 low, 50–100 moderate, ≥100 high), and procedures included Cohen cross‐trigonal and Lich‐Gregoir open reimplantation techniques. Catheterization, stenting and antibiotic prophylaxis protocols (ranging from ≤7 days to up to 3 months) varied by centre and clinical context. Preoperative resistance to one or more antibiotic classes was also evaluated.

A renal sum score (SUMSCR) was developed from preoperative imaging findings (cortical defect, acute pyelonephritis and scarring), assigning a value of 1 for the presence of each condition and 0 for its absence, assuming equal weights.

The model is intended for use during counselling and surgical planning, incorporating available preoperative predictors, while procedural and postoperative data need to be proposed to optimize UTI outcomes.

### 2.4 Model specification, performance and validation

The predictive model for postoperative UTI was developed using a parametric Weibull Accelerated Failure Time (AFT) regression with clustered standard errors to account for centre‐level clustering. Internal validation was guaranteed by bootstrapping and 10‐fold cross‐validation (Table S1 for the model development checklist). Outliers were managed using truncation. Two types of analyses were conducted: an analysis using imputed data for all 404 patients, and a complete‐case analysis including 233 patients with no missing data. Missing data (up to 41.8% for antibiotic variables) were handled via multiple imputation, ensuring rigorous and accurate predictions.

Four models were fitted in each analysis: two full models (all predictors) and two Least Absolute Shrinkage and Selection Operator (LASSO) models (stable predictors), with and without scaling. LASSO shrinks less informative coefficients toward zero, enabling selection of important predictors and regularization to improve model stability and interpretability. The non‐scaled LASSO model, in which predictors were kept in their original clinical units, was chosen as the final model for its robustness and clinical relevance.

Harrell's and Uno's C‐indices (> 0.7 for good discrimination),^26^, ^27^ stratification of predicted risks into quartiles, calibration slope (ideal = 1) and decision curve analysis (DCA) were used to evaluate the model's performance. The net benefit of using the prediction model was compared to performing VCUG in all UTI cases.^28^

### 2.5 Post‐hoc power analysis

A post‐hoc power analysis using G*Power (version 3.1.9.4) indicated that the imputed dataset (n = 404) had 80% power (α = 0.05) to detect effect sizes of R^2^ = 0.0552 (full model, 21 predictors) and R^2^ = 0.0455 (LASSO, 13 predictors). The complete‐case dataset (n = 233) was powered only for larger effects: R^2^ = 0.0988 (full model) and R^2^ = 0.0476 (LASSO, 3 predictors).

### 2.6 Statistical analysis

Analyses were conducted using R software, Vienna, Austria (version 4.4.1), with significance set at p < 0.05. Continuous data were summarized as medians with interquartile ranges (IQR), and categorical data as counts with percentages. Normality was assessed using the Shapiro–Wilk test. Group differences in postoperative UTI were evaluated using Pearson's chi‐squared or Wilcoxon rank‐sum tests, as appropriate. Univariable and multivariable Weibull regressions, including full and LASSO (scaled and non‐scaled) models, were performed to identify risk factors for postoperative UTI.

Scaled models were used to standardize continuous predictors, facilitating comparison and interpretation of effect sizes.

Predictive modelling was performed using both the imputed dataset and the complete‐case dataset for sensitivity analyses. Multiple imputation (m = 10 datasets) was conducted under the missing‐at‐random assumption using the (mice) package, with estimates pooled using Rubin's rules. Variables were imputed by type, predictive mean matching (PMM) for numeric, logistic regression for binary and polytomous regression for categorical, using all predictors to preserve data structure.

A nomogram was generated to estimate the probability of UTI events at 1 and 3 years, enabling individualized risk calculation for new patients. Decision curve analyses were plotted for both time points. Cumulative incidence functions and 1‐minus Kaplan–Meier curves with log‐rank tests were used to illustrate postoperative UTI incidence in the overall cohort and across quartiles of predicted risk and to stratify time to VUR recurrence by postoperative UTI status.

## 3 RESULTS

### 3.1 Baseline characteristics

The study included 404 children, of whom 233 were in the complete‐case dataset, where missing data were limited to four variables (Table 1). The distributions of variables are broadly similar between the imputed and complete‐case datasets. Overall, 56.9% were male, with a median age of 8 years and a BMI of 14.1 kg/m^2^. The median follow‐up was 2.3 years (range 2.0–3.3). Most children had VUR grade IV‐V (95.1%), and 10.6% had received prior endoscopic injection. A median of 4 preoperative febrile UTIs was reported, with 42.3% experiencing fever ≥38°C and 33.9% having renal scarring. Surgery indications included repeated breakthrough UTIs (52.5%) and symptomatic high‐grade VUR (32.2%). Procedures were evenly split between Cohen (47%) and Lich‐Gregoir (53%) techniques. The median operative time was 80 minutes, with 4‐days catheterization and hospitalization. Stents were placed in 33.4%. Antibiotic resistance was common (74.5%), with 23% resistant to >2 classes. Postoperative antibiotics were given for a median of 2 days. Predictor comparisons between children who did and did not develop postoperative UTIs are also shown (Table 1). Age, BMI, reflux phase, prior APN and UTI, preoperative fever, catheter and stent use and antibiotic resistance all affected UTI recurrence (all p < 0.05).

**TABLE 1:** Baseline demographic characteristics and potential clinical, procedural and antibiotic‐related predictors of postoperative UTI in the original cohort of 404 children with dilating VUR and preoperative febrile UTIs.

Characteristics	Overall (n = 404)	Missing, n (%)	Postoperative UTI (n = 111)	P value^a^
*Follow‐up duration, years*	2.3 (2–3.3) max. 11.2		2.1 (2–3) max. 10.2	
Age^b^ at surgery, years	8 (6–9)		8 (7–9)	**0.005**
BMI, kg/m2	14.1 (13.6–17.9)		13.7 (13.4–14.3)	**<0.001**
Sex				0.876
Male	230 (56.9)		62 (55.9)	
Female	174 (43.1)		49 (44.1)	
Prior injection	43 (10.6)		19 (17.1)	**0.016**
Surgical indications				0.308
Repeated breakthrough UTI	212 (52.5)		65 (58.6)	
Symptomatic high‐grade reflux (III‐V)^c^	130 (32.2)		32 (28.8)	
Others^d^	62 (15.3)		14 (12.6)	
Preop VUR Grade				0.396
III	20 (5.0)		3 (2.7)	
IV	124 (30.7)		33 (29.7)	
V	260 (64.4)		75 (67.6)	
Preop VUR phase on VCUG				**<0.001**
At voiding	226 (55.9)		80 (72.1)	
At filling	69 (17.1)		26 (23.4)	
At filling and voiding	109 (27.0)		5 (4.5)	
Ureteral dilation ≥ 7 mm on VCUG	309 (76.5)		81 (73)	0.372
Number of preop febrile UTIs	4 (3–5)	1 (0.2)	5 (4–6)	**<0.001**
Preoperative fever				**0.003**
37.5–37.9°C	233 (57.7)		30 (27)	
38–39°C	84 (20.8)		12 (10.8)	
≥39°C	87 (21.5)		0 (0)	
Preop cortical defect	96 (23.9)	3 (0.7)	3 (2.7)	**<0.001**
Preop acute pyelonephritis	31 (9.5)	76 (18.8)	2 (1.8)	**<0.001**
Preop renal scarring	137 (33.9)		12 (10.8)	**<0.001**
Surgeon experience				0.687
≥100 operations	84 (20.8)		25 (22.5)	
50–100 operations	268 (66.3)		70 (63.1)	
<50 operations	52 (12.9)		16 (14.4)	
Treatment group				0.085
Cohen group	190 (47.0)		44 (39.6)	
Lich‐Gregoir group	214 (53.0)		67 (60.4)	
Operative time, minutes	80 (70–111)		79 (69–140)	0.337
Urethral catheterization duration, days	4 (1–5)		3 (1–4)	**<0.001**
Ureteral stenting	135 (33.4)		13 (11.7)	**<0.001**
Hospitalization time, days	4 (2–5)		3 (2–5)	**0.002**
Preop antibiogram resistance pattern		169 (41.8)		**0.002**
No resistance	60 (25.5)		20 (18)	
Resistance to one antibiotic class	79 (33.6)		27 (24.3)	
Resistance to two classes	42 (17.9)		10 (9)	
Resistance to more than two classes	54 (23.0)		17 (15.3)	
Postop antibiotic duration, days	2 (1–3)	74 (18.3)	2 (1–2)	0.919

*Note*: Data are presented as n (%) or median (IQR); max.: maximum; VCUG: Voiding Cystourethrogram.

^a^ Reported p‐values reflect comparisons between children who developed and did not develop postoperative UTIs for each predictor group.

^b^ With 47 (11.6%) non‐toilet‐trained (< 3 years).

^c^ Reflux associated with recurrent UTIs, renal damage, hydronephrosis or pain.

^d^ Other surgical indications including poor compliance to medical treatment, children with ineffective or poorly tolerated continuous antibiotic prophylaxis, girls with persistent VUR after puberty, VUR recurrence after endoscopic injection, reflux nephropathy, presence of new renal scars on DMSA, worsening in renal function, parental preference, and persistent reflux >2 years.

### 3.2 Postoperative febrile UTI recurrence

In the imputed dataset, febrile UTI occurred in 111 children after the first postoperative month, with cumulative incidences of 26.5% (95% CI: 22.2–30.8) at 1 year and 27.2% (95% CI: 22.9–31.6) at 3 years (Figure S1A). Risk varied substantially across children, with those in the highest risk group having a recurrence rate of 41.6%, compared to much lower rates in the lowest risk group (p < 0.001; Figure S1B). In the complete‐case dataset, febrile UTI occurred in 73 children, but cumulative incidences were not calculated.

### 3.3 Risk factors for postoperative febrile UTI: primary and sensitivity analyses

In univariable analyses of the imputed dataset, older age (HR 3.51, p < 0.001), prior endoscopic injection (HR 1157, p = 0.006), more preoperative febrile UTIs (HR 17.6, p < 0.001) and the Lich–Gregoir technique (HR 83.26, p = 0.022) increased postoperative febrile UTI risk, whereas lower BMI (HR 0.10, p < 0.001), longer catheterization (HR 0.16, p < 0.001) and prolonged hospitalization (HR 0.12, p < 0.001) reduced risk. After adjustment, only preoperative febrile UTIs remained a predictor (non‐scaled HR 3.57, 95% CI 1.14–11.21; scaled HR 9.25, 95% CI 1.25–68.5, p = 0.029). These associations were not confirmed in complete‐case full models, and both models showed overfitting (Tables 2, S2 and S3).

**TABLE 2:** Univariable and multivariable Weibull regression hazard ratios in the full and LASSO non‐scaled models for risk factors of postoperative UTI recurrence based on imputed data (n = 404, including 111 postoperative UTI events) and complete‐case analysis (n = 233, including 73 postoperative UTI events).

Predictors		Univariable			Full Multivariable^a^			LASSO Multivariable^b^					
		Imputed data (n = 404)			Imputed data (n = 404)			Imputed data (n = 404)			Complete‐case (n = 233)		
		HR	95% CI	P value	HR	95% CI	P value	HR	95%CI	P value	HR	95%CI	P value
Age at surgery, years	Non‐scaled Scaled	3.51 26.17	1.73–7.11 4.18–163	***<0.001***									
BMI, kg/m2	Non‐scaled Scaled	0.10 0	0.04–0.23 0–0.01	***<0.001***									
Preop febrile UTIs, n	Non‐scaled Scaled	17.6 150.22	6.68–46.22 27.67–815	***<0.001***	3.57 9.25	1.14–11.21 1.25–68.46	***0.029***						
Prior injection, yes		1157	7.9–169 641	***0.006***							2.08	1.88–2.30	***<0.001***
Treatment, Lich‐Gregoir		83.26	1.87–3706	***0.022***									
Operative time, minutes	Non‐scaled Scaled							1.10 23.13	1.03–1.16 3.06–174	***0.002***	1.007 1.25	1.004–1.01 1.16–1.34	***<0.001***
Catheterization duration, days	Non‐scaled Scaled	0.16 0	0.08–0.31 0–0.02	***<0.001***									
Hospitalization time, days	Non‐scaled Scaled	0.12 0.03	0.04–0.36 0–0.17	***<0.001***									
Postop antibiotic duration, days	Non‐scaled Scaled										0.81 0.67	0.69–0.97 0.48–0.93	***0.018***

*Note*: A Weibull Accelerated Failure Time (AFT) model with centre‐clustered standard errors, 100 bootstraps, and cross‐validation; HR of pooled results from multiple imputed datasets (n = 10) using Rubin's rules (pooling method); bold p‐values indicate significant risks; scaled and non‐scaled models are separate analyses, with HRs for categorical variables are identical, but those for continuous predictors differ between models; Additional variables not shown were included in the multivariable models but were not statistically significant in univariable or multivariable analyses: sex, surgical indication, preoperative VUR grade and phase on VCUG, ureteral dilation, preoperative fever, renal scarring, surgeon experience, stenting, preoperative antibiogram resistance and postoperative antibiotic duration; complete results are provided in Supplementary materials (Tables S2 and S3).

^a^ Included all 21 predictors.

^b^ Included stable predictors present in ≥70% of bootstrap samples.

In LASSO analyses, imputed data outperformed complete‐case data. Operative time was the sole predictor in the imputed dataset (non‐scaled HR 1.10, 95% CI 1.03–1.16; scaled HR 23.13, 95% CI 3.06–174, p = 0.002). In complete‐case analyses, operative time and prior injection were selected, with prior injection showing the strongest association (HR 2.08, 95% CI 1.88–2.30, p < 0.001), and longer postoperative antibiotic duration reduced risk (non‐scaled HR 0.81, 95% CI 0.69–0.97; scaled HR 0.67, 95% CI 0.48–0.93, p = 0.018). Sex was not associated with UTI recurrence in any model (Tables 2, S2 and S3).

### 3.4 Nomogram for febrile UTI prediction and risk stratification

**FIGURE 1:**
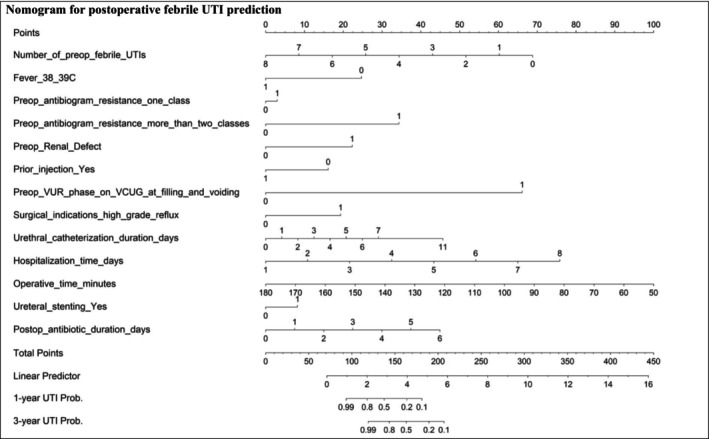
Nomogram for predicting postoperative febrile UTI risk in children with dilating unilateral VUR based on the non‐scaled LASSO model.

**FIGURE 2:**
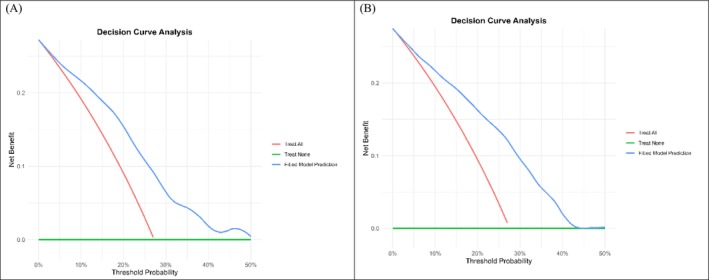
Decision curve analyses (DCA) presented a net benefit in a range of probability between 10% to 40%. (B) and (C): DCA for postoperative UTI at 1‐year and 3‐year follow‐ups, respectively.

A nomogram tool was developed from the non‐scaled LASSO model of the imputed dataset with 13 stable predictors to estimate postoperative UTI risk (Figure 1). This tool demonstrated a strong ability to distinguish between higher‐ and lower‐risk children (Harrell's C‐index = 0.743 ± 0.047; Uno's C‐index = 0.743 ± 0.005), and its predictions closely matched observed outcomes (calibration slope = 1.00 ± 0.29). It is particularly useful for predicting UTI risk in children with expected probabilities between 10% and 40% (Figure 2 and Table S4). Within this range, the tool can guide decisions around VCUG reassessment and specific follow‐up care. However, it is less informative for predicted risks above 40%.

### 3.5 Long‐term VUR recurrence and postoperative febrile UTI

**FIGURE 3:**
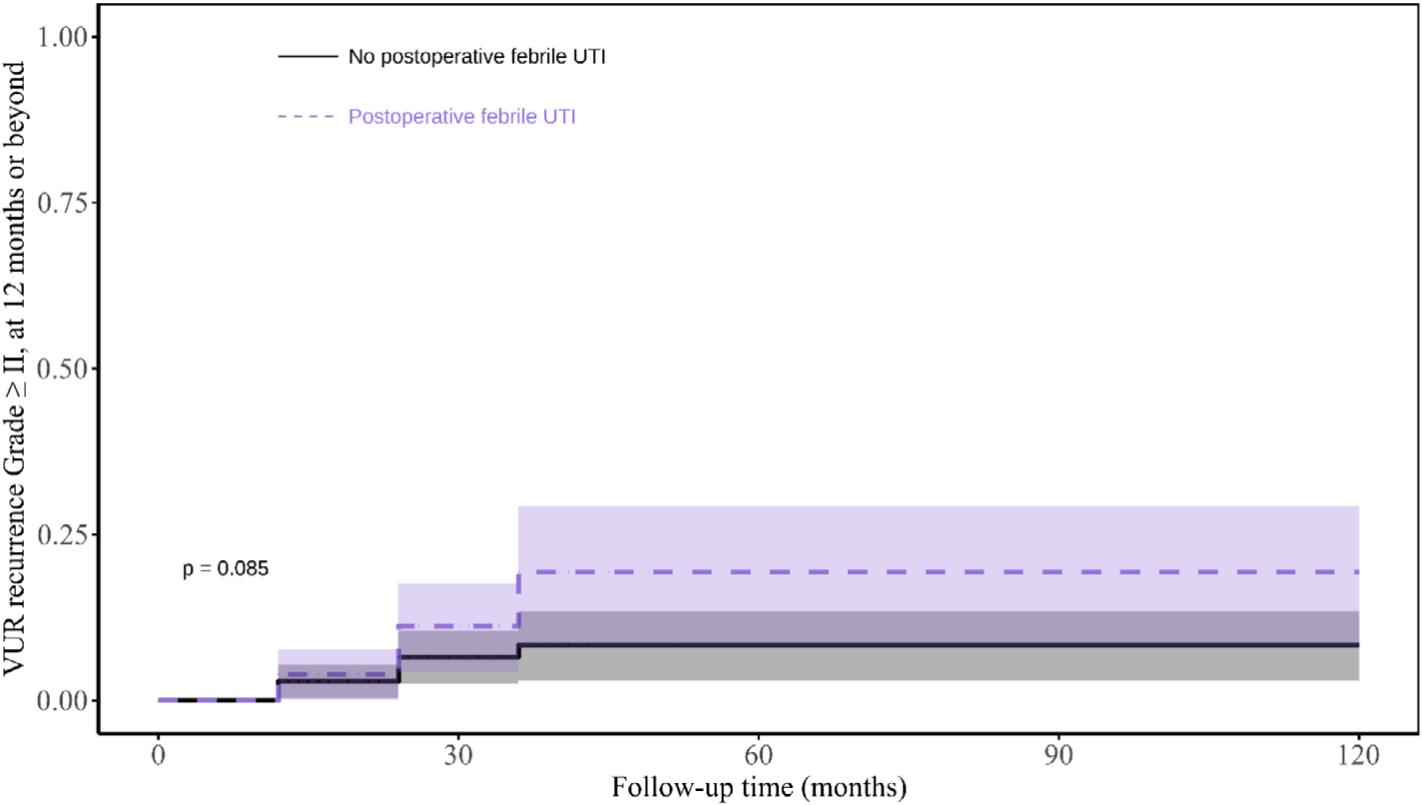
Association between postoperative febrile UTI and VUR recurrence of grade ≥2 at 12 months or beyond, with log‐rank test significance at P < 0.05.

Among children (n = 273, 67.6%) with postoperative VCUG results at 12 months or later, recurrent VUR (grade ≥II) occurred in 3.3% (95% CI: 1.2%–5.4%) at 1 year, increasing to 12.7% (7.2%–17.8%) at 3 years, with no further increase observed up to the maximum follow‐up of 11.2 years. Most recurrent cases (83%) were VUR grade II. Importantly, recurrent VUR did not significantly affect the rate of postoperative UTI (p = 0.085, Figure 3).

## 4 DISCUSSION

Recurrent UTIs are still a major concern in children with VUR following surgery, with rates as high as 40% after reimplantation (Table S5).^6^, ^7^, ^8^, ^9^, ^10^, ^11^, ^12^, ^13^, ^14^, ^15^, ^16^, ^17^ Improving outcomes in high‐risk cases and avoiding unnecessary interventions in low‐risk ones require tailored postoperative care. This study supports personalized risk assessment by identifying recurrence patterns and proposing a predictive model. We excluded children with bowel and bladder dysfunction to avoid the confounding effect of a condition known to independently predispose to recurrent UTIs, even after successful reimplantation.^12^ This allowed for a clearer assessment of postoperative risk factors within a more uniform population of children with normal voiding and primary dilating VUR.

The study found that within the first year after surgery, about one out of four children (26.5%) with unilateral dilating VUR developed postoperative febrile UTIs, with similar rates persisting over three years. These rates are consistent with reported ranges, though they are toward the higher end. However, direct comparisons are limited by differences in cohort and outcome definitions (Table S5). Dogan et al.^12^ reported a 27.2% recurrence of UTIs in 398 children with VUR (grades I–V, unilateral and bilateral) at a median age of 4.9 years, including any postoperative UTI. Oberson et al.^16^ found a 23% recurrence in a younger cohort (N = 130, median age 2.6 years) using a broader febrile UTI definition (<38.5°C). Nelson et al.^17^ observed a 21.8% overall UTI rate, with febrile UTIs accounting for only 6.3% among 1076 children with all‐grade VUR at a median age of 5 years. These variations likely reflect differences in reflux grade, laterality and fever definitions, with our findings suggesting a higher risk of febrile UTI (≥ 38°C) in unilateral dilating VUR.

Most UTI recurrences in our cohort occurred within the first year after surgery (1 month–1 year), indicating a higher risk during this period. Infections might occur after the first year following surgery, but at a lower risk. These findings highlight the value of ongoing surveillance, particularly in the first postoperative year, to improve infection control and to prevent further renal damage. Notably, catheters and stents were typically removed within the first month, and only one febrile UTI was linked to late removal. Excluding early postoperative UTIs and asymptomatic bacteriuria may partly explain the limited influence of early indwelling devices on recurrence patterns. Interestingly, longer catheterization and hospitalization were associated with a reduced risk of UTI in the univariable analysis. However, these and other univariable associations did not remain significant after adjustment, likely reflecting confounding by patient complexity, surgical technique or institutional practices, or, in the complete‐case analysis, with the smaller sample size limiting power to detect smaller effects.

Through full multivariable analyses, each additional preoperative febrile UTI increased risk fourfold (HR 3.57, p = 0.029), with cumulative risk rising for children with multiple infections above the mean (HR 9.25, p = 0.029; equivalent to 1 SD above the mean, mean = 4.4 UTIs, SD ≈ 2). Thus, children with frequent preoperative febrile UTIs are at higher risk and require careful pre‐ and postoperative management. However, the effect was unstable, with evidence of overfitting, and was not confirmed in complete‐case or LASSO analyses. Nevertheless, this finding is consistent with Nelson et al.,^17^ who reported a similar association with postoperative pyelonephritis, a more severe form of infection involving the kidneys. In this cohort, 58.6% of all postoperative UTIs occurred in patients with repeated breakthrough infection indications. While surgical indication was not significantly associated with recurrence, this subgroup's contribution suggests that a higher preoperative infection burden may cause persistent urinary tract changes, increasing future risk.

In the robust imputed LASSO models, which use penalization to select key predictors, longer operative time was identified as an independent risk factor for postoperative UTI, with each additional minute increasing the risk by 10% (HR 1.10, p = 0.002) and each half‐hour above the mean of 95 minutes (≈1 SD, 34 minutes) increasing the risk approximately 23‐fold (HR 23.13, p = 0.002). As hazard ratios multiply, this finding suggests that prolonged surgeries may carry a substantially higher UTI risk than minute‐by‐minute estimates suggest, emphasizing the potential value of optimizing operative efficiency. Consistent with these findings, operative time emerged as a risk factor only when certain patient characteristics, surgeon experience and treatment techniques were excluded from the model, and it was correlated with several clinical and procedural variables (data not shown). This observation suggests that operative time may act as a composite marker of patient complexity and procedure‐related factors, including differences in learning curve and inherent variations between surgical techniques. It may also capture unmeasured intraoperative factors, such as workflow inefficiencies or poor team coordination. Biologically, longer procedures may involve greater tissue manipulation, leading to inflammation and devascularization, which weaken local defences against bacterial colonization and facilitate pathogen entry. Therefore, the risk of infection may be decreased by cutting down on the length of surgery through coordinated efforts on multiple fronts.

In the complete‐case LASSO models, operative time remained a risk factor, despite mild overfitting. Prior endoscopic injection was also associated with an approximately twofold increased risk, while longer postoperative prophylaxis duration reduced the risk by about 20% per additional day (HR 0.81, p = 0.018). These findings suggest that both procedural factors and postoperative management may influence UTI risk, although some caution is warranted given the model's limitations.

No association was observed between sex and postoperative UTI recurrence in this study, even though female sex is a known risk factor in the paediatric population.^12^ The cohort included more males than females (56.9% vs. 43.1%, p = 0.004), with similar median ages but longer follow‐up among males (Table S6). The survival model inherently accounted for follow‐up differences, but the unbalanced sex distribution and incomplete circumcision data (about half of boys circumcised, one‐third missing) may have attenuated potential differences. Circumcision was excluded from analysis as its cultural determination made the missing values not at random and unsuitable for imputation. Larger or more balanced cohorts with complete circumcision data may be needed to clarify sex differences in postoperative UTI risk.

In this cohort, 74.5% of preoperative infections were caused by antibiotic‐resistant organisms, and this subgroup accounted for 73% of postoperative UTIs with available resistance data. Such resistance likely increases postoperative recurrence risk, even though not demonstrated here, possibly due to persistent colonization. This aligns with a recent review showing that patients on continuous prophylaxis are more prone to breakthrough infections from resistant uropathogens,^29^ and we believe this association may persist postoperatively in children with prior resistance. Moreover, resistance may limit postoperative prophylaxis effectiveness by narrowing antibiotic options and shortening duration. Indeed, 75% of patients received TMP‐SMX or nitrofurantoin for only 1–3 days. Rigorous studies with detailed microbiological and resistance data are warranted to assess whether longer or targeted prophylaxis could reduce UTI recurrence after surgery. If confirmed, these findings would emphasize the need for culture‐guided antibiotic selection and vigilant postoperative care in children with resistant infections.

Of particular importance, this study informed a novel predictive nomogram for postoperative UTI recurrence, which showed strong discrimination within the 10%–40% risk range. This nomogram serves as a decision‐support tool that promotes a more individualized approach to risk by acknowledging that different people have different perceptions about what constitutes an “acceptable” risk, beyond the commonly cited 5% missed diagnosis rate. This makes it easier for families and clinicians to make shared decisions by balancing the risks of missed recurrence against the potential harms of unnecessary procedures. Follow‐up control VCUG, for example, can be tailored for each child based on predicted risk. However, this nomogram was not very helpful for children at higher risk (> 40%), which emphasizes the need to reevaluate management strategies for this subgroup.

For a more complete assessment of postoperative infection risk, the study found VUR recurrence rates (grade ≥ II) of 3.3%–12.7% at 1–3 years, with no significant association with postoperative UTI. This indicates that reflux resolution alone does not reliably predict postoperative infection, emphasizing the value of the nomogram as a tool to help guide effective follow‐up strategies. Similarly, Hubert et al.,^30^ found no significant difference in febrile UTI recurrence despite higher rates in children with persistent reflux (11.9%) compared to those with resolved reflux (6.6%). The markedly higher prevalence of high‐grade (IV‐V) reflux in our cohort (95% vs. 1%) further supports that the lack of a UTI–VUR recurrence association persists across all reflux grades.

As far as we know, this is the first study to develop a prognostic model for postoperative UTI recurrence. Its multicentred design, robust and novel statistical analyses and sensitivity analyses strengthened the model's accuracy and maintained its practical applicability in clinical settings.

However, certain limitations should be acknowledged. First, the retrospective design introduces inherent biases. Although the model showed strong internal performance, it was evaluated only on the available dataset without external validation. Selection bias may limit its applicability, making it more relevant to high‐resource settings, such as tertiary paediatric centres, and less so to low‐resource environments. Variations in surgical technique and care delivery across centres, not fully captured by adjustment, may also affect validity. The generalizability of the absent UTI–VUR association may be limited, as outcomes were based on postoperative VCUG performed for either routine or UTI‐driven screening. Information bias is another concern: censoring, loss to follow‐up and incomplete documentation can compromise long‐term UTI assessment. In practice, missing data and underreporting can result from inconsistent follow‐up, patient migration or delays in UTI documentation. To mitigate this, UTI outcomes were reported at 1‐ and 3‐year intervals. Additionally, infections occurring within the first 30 postoperative days were not collected. While these are often transient and perioperative, their exclusion may underestimate early postoperative UTIs. Incomplete circumcision data among males, which could not be reliably imputed, may have also limited the assessment of sex‐related differences. Furthermore, imputation of missing antibiotic data may have influenced associations between antibiotic exposure and postoperative UTI, though UTI outcomes themselves were not imputed. Lastly, residual confounding from unmeasured factors, particularly related to pathogens and antibiotic use, may persist and should be explored in future studies.

## 5 CONCLUSIONS

In children with primary unilateral dilating VUR treated with open reimplantation, reflux recurrence was low, but postoperative febrile UTIs remained a clinically significant concern with a rate exceeding 27% over 3 years. This study developed a nomogram that stratifies children at high and low risk profiles for postoperative UTIs. It also supports shared decisions on follow‐up interventions, such as control VCUG, for infection risks between 10% and 40%, based on risk tolerance. This tool still requires external validation before broader application. Prospective studies can also refine it and enhance its clinical impact as our understanding of UTI risk factors advances.

## AUTHOR CONTRIBUTIONS

*Conception and design:* Khadija Ismail, Mohamad Moussa, Bilal Aoun and Mohamad Abou Chakra. *Acquisition of data:* Khadija Ismail, Bilal Aoun, Mohamad Abou Chakra, Anthony Kallas‐Chemaly, Priyank Yadav, Christian Kruppa, Katrin Schuchardt and Alexandra Wilke. *Analysis and interpretation of data:* Khadija Ismail and Mohamad Abou Chakra. *Drafting of the manuscript:* Khadija Ismail. *Critical revision of the manuscript for important intellectual content:* Mohamad Moussa, Bilal Aoun, Mohamad Abou Chakra, Anthony Kallas‐Chemaly, Priyank Yadav, Christian Kruppa, Katrin Schuchardt, Alexandra Wilke, Pascale Salameh and Amal Al‐Hajje. *Statistical analysis:* Khadija Ismail. *Supervision:* Mohamad Moussa, Bilal Aoun, Pascale Salameh and Amal Al‐Hajje.

## CONFLICT OF INTEREST STATEMENT

The authors have no conflicts of interest related to the work reported in this paper.

## ETHICS STATEMENT

This research involving human data has been approved by the Institutional Review Board of three study centres: IRB ID BIO‐2022‐0108 from American University of Beirut Medical Centre, Beirut, Lebanon; IRB No. CEHDF 2118 from Hôtel Dieu de France, Beirut, Lebanon; and Reference No. 3/2022 from Al‐Zahraa University Medical Centre, Beirut, Lebanon. Additionally, verbal data‐sharing agreements were established with Sanjay Gandhi Postgraduate Institute of Medical Sciences in India and University Hospital Carl Gustav Carus in Germany.

Informed consent was waived by the IRB (ID BIO‐2022‐0108) of the American University of Beirut Medical Centre due to minimal risk, use of existing records without direct contact and impracticability of obtaining consent from a large, hard‐to‐contact paediatric population and their guardians.

## Supporting information


**Figure S1.** Cumulative incidence probability of postoperative urinary tract infection (UTI) events. (A) and (B): Probability in the entire cohort of children with dilating unilateral vesicoureteral reflux and preoperative UTIs, and according to quartiles of the pooled linear predictor resulting from the non‐scaled LASSO AFT Weibull model with clustered standard errors, respectively.
**Table S1.** Checklist for the predictive model design.
**Table S2.** Univariable Weibull regression hazard ratios for the scaled and non‐scaled risk factors of postoperative UTI recurrence based on imputed data (n = 404, including 111 postoperative UTI events) and complete‐case analysis (n = 233, including 73 postoperative UTI events).
**Table S3.** Multivariable Weibull regression hazard ratios in the full and LASSO scaled and non‐scaled models for risk factors of postoperative UTI recurrence based on imputed data (n = 404, including 111 postoperative UTI events) and complete‐case analysis (n = 233, including 73 postoperative UTI events).
**Table S4.** Net benefit for performing postoperative VCUG in all children who developed UTIs 1‐year after surgery or according to the prediction model using a threshold probability of pt.
**Table S5.** Characteristics and results of previous studies reporting radiological and clinical success outcomes of children with VUR treated with open Cohen or Lich‐Gregoir techniques in reverse chronological order.
**Table S6.** Age and follow‐up duration comparison by sex.

## Data Availability

The data and analysis code are available in the following GitHub repository: https://github.com/khadija315/UTI-risk-prediction/tree/74ccff5da164f0aef316e6ca4fcc5760c570375a
