# Supplementary material for: Development of a prediction model for urinary tract infection risk after open reimplantation in children with primary unilateral vesicoureteral reflux: A multicentre study
Source: BJUI Compass. 2025 Nov 17;6(11):e70111. doi: 10.1002/bco2.70111 (PMC12623004; doi:10.1002/bco2.70111)
Supplement: Supplementary file 1 — Figure S1. Cumulative incidence probability of postoperative urinary tract infection (UTI) events. (A) and (B): Probability in the entire cohort of children with dilating unilateral vesicoureteral reflux and preoperative UTIs, and according to quartiles of the pooled linear predictor resulting from the non‐scaled LASSO AFT Weibull model with clustered standard errors, respectively. Table S1. Checklist for the predictive model design. Table S2. Univariable Weibull regression hazard ratios for the scaled and non‐scaled risk factors of postoperative UTI recurrence based on imputed data (n = 404, including 111 postoperative UTI events) and complete‐case analysis (n = 233, including 73 postoperative UTI events). Table S3. Multivariable Weibull regression hazard ratios in the full and LASSO scaled and non‐scaled models for risk factors of postoperative UTI recurrence based on imputed data (n = 404, including 111 postoperative UTI events) and complete‐case analysis (n = 233, including 73 postoperative UTI events). Table S4. Net benefit for performing postoperative VCUG in all children who developed UTIs 1‐year after surgery or according to the prediction model using a threshold probability of pt. Table S5. Characteristics and results of previous studies reporting radiological and clinical success outcomes of children with VUR treated with open Cohen or Lich‐Gregoir techniques in reverse chronological order. Table S6. Age and follow‐up duration comparison by sex. [file BCO2-6-e70111-s001.pdf]

Title

Development of a prediction model for urinary tract infection risk after open reimplantation in children with primary unilateral vesicoureteral reflux: A multicentre study

Supplementary materials

Table of contents

Figure S1..2

Table S1...3
Table S2...4
Table S3...5
Table S4...6
Table S5...7
Table S6...9

Supplementary Materials

(A)

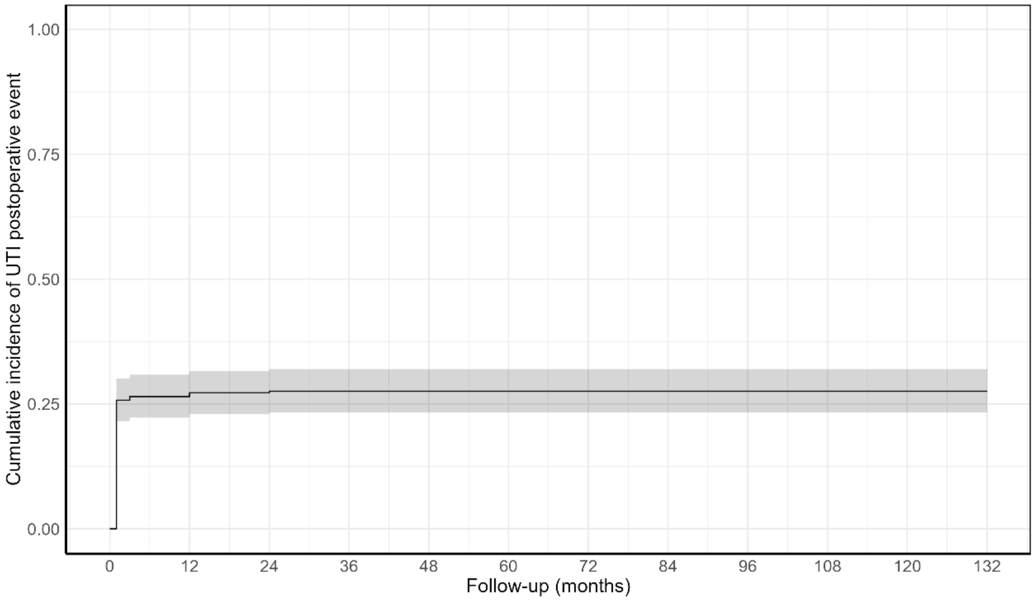

(B)

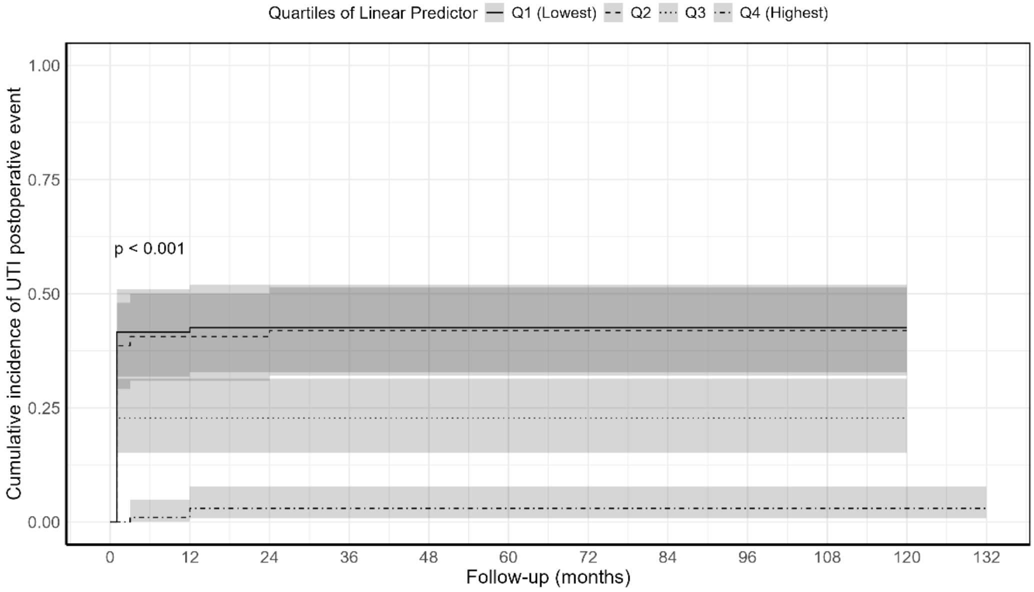

Figure S1

Cumulative incidence probability of postoperative urinary tract infection (UTI) events.

(A) and (B): Probability in the entire cohort of children with dilating unilateral vesicoureteral reflux and preoperative UTIs, and according to quartiles of the pooled linear predictor resulting from the non-scaled LASSO AFT Weibull model with clustered standard errors, respectively

Table S1

Checklist for the predictive model design.

Steps	Predictive model
General considerations	
Research question	Emphasis on prediction
Intended application	- One reasonable strategy in the postoperative UTI risk study would be to reevaluate VUR resolution radiographically through VCUG when UTI occurs (vs. no UTI - no VCUG), and to follow up a specific care regimen (in timing: early/late, quality: conservative/aggressive). This study adds to the above reasonable strategy a risk stratification, it relates this strategy to a specific category of patients (risk profile).<br>- The intended use of the predictive model is during patient counseling and surgical planning, where demographic, clinical, and preoperative antibiotic-related predictors are available, while procedural and postoperative antibiotic-related predictors need to be determined to optimize UTI outcomes.
Outcome	We estimated the 1- and 3-year postoperative UTI recurrence risks.
Predictors	Predictors included demographic, clinical, and procedural factors, preoperative antibiotic resistance and postoperative antibiotic duration.<br>They are divided into available (preoperative) and modifiable (procedural/postoperative).
Study design	Retrospective multicenter cohort
Statistical model	Weibull Accelerated Failure Time (AFT) regression for long-term outcome with center clustering, LASSO regularization, non-scaled variables (main model), bootstrapping (100 samples), and 10-fold cross-validation
Sample size	404 children, 111 UTI events<br>Using linear multiple regression on G*Power version 3.1.9.4 (fixed model, R2 deviation from zero), a sample of 404 children was adequate to detect an effect size of R2 = 0.0552 (full model, 21 predictors) and R2 = 0.0455 (LASSO model, 13 predictors) with $\alpha = 0.05$ and 80% power.
Seven modelling steps	
Data inspection	Outliers: Truncation of continuous predictors using interquartile range (IQR) or percentile rules.<br>Missing values: multiple imputation with 10 datasets and 50 iterations.<br>Imputation used predictive mean matching for numeric variables, logistic regression for binary variables, and polytomous regression for categorical variables.
Coding of predictors	Categorical predictors:<br>- Combining levels to limit the degrees of freedom of predictors in a model (e.g. surgical indications)<br>- Creating sum score (SUMSCR, preoperative renal status)<br>All categorical variables were treated as dummy variables in Weibull regression model.
Model specification	Penalized estimation with LASSO (shrinkage to 0)<br>Clustering by centers
Model estimation	- Regression coefficients (Hazard ratios with 95% CI)<br>- Linear predictors
Model performance	Optimism-corrected performance: Harrell's C-index , Uno's C-index, calibration, clinical utility via decision curve analysis (DCA)
Model validation:<br>Stability	Internal validation: Bootstrap* and cross-validation<br>No external validation<br>*Stable predictors in the LASSO model were those selected in ≥70% of bootstrap samples.<br>Model development and validation used the following R libraries: (survival), (glmnet), (sandwich), (lmtest), (dplyr), (mitools), (rms), (dcurves), (tidyverse), (cmprsk), (tidycmprsk), and (ggsurvfit).
Model presentation	Nomogram
Validity	
Internal: Overfitting	Attempts to limit and correct for overfitting: large sample size, predictors from literature, LASSO for selection and shrinkage
External: Generalizability	- The predictions can reasonably apply to populations that share similar characteristics to the one studied. However, the population treated surgically can slightly differ from the study population.<br>- The predictions in high-resource hospitals may be valid for similar pediatric populations in other high-resource settings. However, it may not be valid in low-resource settings where care delivery differs significantly.

Table S2

Univariable Weibull regression hazard ratios for the scaled and non-scaled risk factors of postoperative UTI recurrence based on imputed data (n = 404, including 111 postoperative UTI events) and complete-case analysis (n = 233, including 73 postoperative UTI events).

**Predictors**		**Univariable**					
		**Imputed Data (n=404)**			**Complete-Case (n=233)**		
		**HR**	**95% CI**	***p***	**HR**	**95%CI**	***p***
Age at surgery, years	**Non-scaled**	3.51	1.73–7.11	***<0.001***	1.07	0.84–1.37	*0.562*
	**Scaled**	26.17	4.18–163	***0.001***	1.20	0.64–2.25	*0.562*
BMI, kg/m2	**Non-scaled**	0.10	0.04–0.23	***<0.001***	0.82	0.65–1.03	*0.092*
	**Scaled**	0	0–0.01	***<0.001***	0.51	0.24–1.12	*0.092*
Sex (Female)		2.70	0.06–114.5	*0.603*	0.81	0.43–1.52	*0.516*
Prior injection (Yes)		1157	7.9–169641	***0.006***	1.99	0.98–4.03	*0.056*
Surgical indications							
Others		1			1		
Repeated breakthrough UTI		5.79	0.009–3373	*0.112*	1.11	0.38–3.26	*0.844*
Symptomatic high-grade reflux (III-V)		118	0.33–42634	*0.589*	0.81	0.31–2.12	*0.664*
Preop VUR Grade							
III		1			1		
IV		4775488	0–Inf	–	862	0–6x10^{9}	*0.402*
V		9235578	0–Inf	–	958	0–8x10^{9}	*0.399*
Preop VUR phase on VCUG							
At voiding		1			1		
At filling		1.65	0.04–65.06	*0.789*	0.90	0.50–1.64	*0.736*
At filling and voiding		0	0–Inf	–	0.0002	0–2917	*0.320*
Ureteral dilation≥ 7mm on VCUG		0.06	0–3.67	*0.176*	1.22	0.57–2.61	*0.612*
Number of preop febrile UTIs	**Non-scaled**	17.6	6.68–46.22	***<0.001***	1.09	0.79–1.51	*0.607*
	**Scaled**	150.22	27.67–815	***<0.001***	1.16	0.66–2.05	*0.607*
Preoperative fever							
37.5-37.9°C		1			1		
38-39°C		8.63	0.15–486.6	*0.295*	1.03	0.40–2.65	*0.955*
≥39°C		0	0–0.02	***0.001***	0.61	0.01–29.78	*0.801*
Sumscore (SUMSCR)							
No renal scarring		1			1		
Any renal defect on DMSA		3.4x10^{-07}	1.9x10^{-09}–6.2x10^{-05}	***<0.001***	0.45	0.12–1.68	*0.233*
Surgeon experience							
≥100 operations		1			1		
50 - 100 operations		0.18	0–17.26	*0.457*	0.89	0.40–1.98	*0.782*
<50 operations		0.46	0–311.3	*0.815*	1.18	0.54–2.58	*0.685*
Treatment group							
Cohen group		1			1		
Lich-Gregoir group		83.26	1.87–3706	***0.022***	0.81	0.39–1.66	*0.561*
Operative time, minutes	**Non-scaled**	1.04	0.98–1.1	*0.157*	1.01	1.00–1.02	*0.094*
	**Scaled**	4.01	0.59–27.35	*0.157*	1.28	0.96–1.72	*0.094*
Urethral catheterization duration, days	**Non-scaled**	0.16	0.08–0.31	***<0.001***	0.93	0.77–1.13	*0.471*
	**Scaled**	0	0–0.02	***<0.001***	0.78	0.39–1.54	*0.471*
Ureteral stenting (Yes)		0	0–0		0.57	0.09–3.65	*0.550*
Hospitalization time, days	**Non-scaled**	0.12	0.04–0.36	***<0.001***	1.00	0.84–1.20	*0.991*
	**Scaled**	0.03	0–0.17	***<0.001***	1.00	0.73–1.37	*0.991*
Preop antibiogram resistance pattern							
No resistance		1			1		
Resistance to one antibiotic class		3.84	0.05–298.6	0.544	1.03	0.46–2.31	0.938
Resistance to two classes		0.01	0–3.1	0.113	0.56	0.14–2.18	0.399
Resistance to more than two classes		0	0–0.06	**0.003**	0.96	0.43–2.14	0.922
Postop antibiotic duration, days	**Non-scaled**	0.74	0.29–1.88	0.526	0.83	0.64–1.07	*0.154*
	**Scaled**	0.55	0.08–3.56	0.526	0.69	0.42–1.15	*0.154*

A Weibull Accelerated Failure Time (AFT) model with center-clustered standard errors, 100 bootstraps, and cross-validation; HR of pooled results from multiple imputed datasets (n=10) using Rubin's rules (pooling method); Bold p-values indicate significant risks; "–" indicates unstable estimations; Scaling is a transformation used exclusively for continuous variables

Table S3

Multivariable Weibull regression hazard ratios in the full and LASSO scaled and non-scaled models for risk factors of postoperative UTI recurrence based on imputed data (n = 404, including 111 postoperative UTI events) and complete-case analysis (n = 233, including 73 postoperative UTI events).

**Predictors**		**Full Multivariable [a]**						**LASSO Multivariable [b]**					
		**Imputed Data (n=404)**			**Complete-Case (n=233)**			**Imputed Data (n=404)**			**Complete-Case (n=233)**		
		**HR**	**95%CI**	***p***	**HR**	**95%CI**	***p***	**HR**	**95%CI**	***p***	**HR**	**95%CI**	***p***
Age at surgery, years	**Non-scaled**	0.89	0.37–2.15	*0.795*	0.87	0.58–1.31	*0.511*						
	**Scaled**	0.74	0.07–7.31	*0.795*	0.7	0.24–2.01	*0.511*						
BMI, kg/m2	**Non-scaled**	0.91	0.29–2.9	*0.876*	0.95	0.46–1.96	*0.887*						
	**Scaled**	0.73	0.01–38.61	*0.876*	0.84	0.07–9.85	*0.887*						
Sex (Female)		1.73	0.1–29.03	*0.702*	0.95	0.36–2.52	*0.916*						
Prior injection (Yes)		4.59	0.1-213.67	*0.437*	2.45	0.68–8.89	*0.172*	7.52	0.15–368.99	*0.310*	2.08	1.88–2.30	***<0.001***
Surgical indications													
Others		1			1								
Repeated breakthrough UTI		0.45	0–60.07	*0.752*	0.88	0.12–6.22	*0.899*						
Symptomatic high-grade reflux (III-V)		0.04	0–6.63	*0.219*	0.77	0.09–6.28	*0.804*	0.13	0.01–3.09	*0.205*			
Preop VUR Grade													
III		1			1								
IV		65.13	0–Inf	–	1.04	0–Inf	*1*						
V		25.5	0–Inf	–	0.77	0–Inf	*1*						
Preop VUR phase on VCUG													
At voiding		1			1								
At filling		6.76	0.02–2730	*0.532*	1.19	0.11–12.8	*0.884*						
At filling and voiding		0	0–Inf	–	0	0–Inf	*1*	0	0–7.21	*0.120*			
Ureteral dilation≥ 7mm on VCUG		1.16	0.04–37.66	*0.932*	1.09	0.31–3.83	*0.888*						
Number of preop febrile UTIs	**Non-scaled**	3.57	1.14–11.21	***0.029***	1.00	0.56–1.76	*0.988*	2.65	0.84–8.32	*0.095*			
	**Scaled**	9.25	1.25–68.46	***0.029***	1.00	0.36–2.7	*0.988*	5.49	0.74–40.65	*0.095*			
Preoperative fever													
37.5-37.9°C		1			1								
38-39°C		16.06	0.45–569.03	*0.127*	1.34	0.23–7.8	*0.747*	17.37	0.57–530.04	*0.102*			
≥39°C		5.92	0.04–920.4	*0.490*	0.63	0–Inf	*1*						
Sumscore (SUMSCR)													
No renal scarring		1			1								
Any renal defect on DMSA		0.03	0–6.55	*0.206*	–	–	–	0.05	0–8.45	*0.255*			
Surgeon experience													
≥100 operations		1			1								
50 - 100 operations		26.68	0.06–12529	*0.295*	1.5	0.11–20.02	*0.759*						
<50 operations		40.35	0.02–87521	*0.345*	2.07	0.12–35.34	*0.617*						
Treatment group													
Cohen group		1			1								
Lich-Gregoir group		0.06	0–70.1	*0.435*	0.43	0.02–11.56	*0.617*						
Operative time, minutes	**Non-scaled**	1.07	0.98–1.16	*0.146*	1.01	0.98–1.04	*0.455*	1.10	1.03–1.16	***0.002***	1.007	1.004–1.01	***<0.001***
	**Scaled**	8.77	0.47–163.62	*0.146*	1.48	0.53–4.12	*0.455*	23.13	3.06–174	***0.002***	1.25	1.16–1.34	***<0.001***
Urethral catheterization duration, days	**Non-scaled**	0.59	0.14–2.38	*0.455*	0.88	0.45–1.69	*0.692*	0.55	0.14–2.2	*0.399*			
	**Scaled**	0.16	0–20.22	*0.455*	0.63	0.06–6.18	*0.692*	0.13	0–15.42	*0.399*			
Ureteral stenting (Yes)		0.28	0–61.09	*0.641*	1.18	0.23–6.00	*0.845*	0.59	0–117.49	*0.845*			
Hospitalization time, days	**Non-scaled**	0.21	0.04–1.15	*0.071*	0.74	0.34–1.59	*0.439*	0.27	0.05–1.6	*0.148*			
	**Scaled**	0.06	0–1.27	*0.071*	0.59	0.16–2.24	*0.439*	0.1	0–2.26	*0.148*			
Preop antibiogram resistance pattern													
No resistance		1			1								
Resistance to one antibiotic class		1.85	0.06–60.97	*0.729*	1.34	0.39–4.62	*0.648*	1.96	0.08–50.88	*0.686*			
Resistance to two classes		0.26	0–29.2	*0.573*	1.26	0.22–7.15	*0.796*						
Resistance to more than two classes		0.07	0–5.18	*0.229*	0.82	0.19–3.59	*0.787*	0.13	0–7.32	*0.325*			
Postop antibiotic duration, days	**Non-scaled**	0.39	0.14–1.08	*0.071*	0.85	0.55–1.29	*0.435*	0.4	0.15–1.11	*0.078*	0.81	0.69–0.97	***0.018***
	**Scaled**	0.15	0.02–1.18	0.071	0.72	0.31–1.65	*0.435*	0.16	0.02–1.22	*0.078*	0.67	0.48–0.93	***0.018***
**Harrell's C-index**	***Non-scaled model***	*0.276 ± 0.051*			*0.466 ± 0.09*			*0.743 ± 0.047*			*0.389 ± 0.077*		
**Uno's C-index**		*0.276 ± 0.009*			*0.466 ± 0.046*			*0.743 ± 0.005*			*0.389 ± 0.04*		
**Calibration slope**		*-1.62 ± 0.585 → overfitted model*			*0.235 ± 0.168 → overfitted model*			*1 ± 0.290 → good prediction*			*0.679 ± 0.222 → mild overfitting*		

A Weibull Accelerated Failure Time (AFT) model with center-clustered standard errors, 100 bootstraps, and cross-validation; HR of pooled results from multiple imputed datasets (n=10) using Rubin's rules (pooling method) in the imputed data; [a]Included all 21 predictors; [b]Included stable predictors present in ≥70% of bootstrap samples; Bold p-values indicate significant risks; "–" indicates unstable estimations; Scaled and non-scaled models are separate analyses; HRs for categorical variables are identical, but those for continuous predictors differ between models

Table S4

Net benefit for performing postoperative VCUG in all children who developed UTIs 1-year after surgery or according to the prediction model using a threshold probability of pt.

**For 1-year UTI prediction**	**Net benefit**		**Advantage of model**	
**pt (%)**	Treat all	Prediction Model	Net benefit	Reduction in postoperative VCUG per 100 patients
**10%**	0.138	0.219	0.081	73
**20%**	0.031	0.158	0.127	51
**30%**	-0.108	0.063	0.171	40
**40%**	-0.292	0.019	0.311	47
**50%**	-0.551	0.005	0.556	56

The reduction in the number of unnecessary VCUG after development of postoperative UTIs per 100 patients is calculated as:

$$\frac{net\ benefit\ of\ the\ model - net\ benefit\ of\ treat\ all}{{p_t}/{(1-p_t)}} \times 100$$

Table S5

Characteristics and results of previous studies reporting radiological and clinical success outcomes of children with VUR treated with open Cohen or Lich-Gregoir techniques in reverse chronologic order.

Author, y	N	Age at operation Median (range) /Mean ± SD / Mean	Study design	Study techniques (N)	Preop VUR Grade/Mean ± SD (laterality)	ATB administration Preop / Postop	Follow up duration	VUR resolution (grade 0)	Postop UTI definition	Postop UTI rate/ number
**Tessier et al., 2022**[6]	171 (total)/ 77 Intra	Cohen: mean 3.8 (1 month -11 years)	Multi (3 centers); C; Retro	Intra: Open Cohen<br>Extra: LAP LG<br>Endoscopic injection	Total: III (uni+ bi)	Yes / No	Mean: 5.3 years<br>Range: 2–11 years	–	Febrile UTIs*	Intra: 6.5%
**Aydin et al., 2020**[7]	38 (total)/ 18 Intra + 20 Extra	Intra: 3.4 ± 1.8 years<br>Extra: 5.5 ± 2.9 years	Mono; C; Retro	Intra: Cohen<br>Extra: LG	Total: III-V (uni)<br>Intra: 3.7 ± 0.8 (uni)<br>Extra: 3.9 ± 0.8 (uni)	Yes / –	Intra: 29.9 ± 18.9 months<br>Extra: 28.4 ± 17.3 months	Intra: 100%<br>Extra: 100%	Afebrile cystitis with positive urine culture	Intra: 16.6%<br>Extra: 0%<br>(in up to 1 year)
**Silay et al., 2018**[8]	58 (total)/ 23 Intra + 35 Extra	Intra: 4.6 ± 1.6 years<br>Extra: 7.6 ± 4.2 years	Mono; C; Retro	Intra: Cohen<br>Extra: LG	Total: III-V (uni)<br>Intra: 3.4 ± 0.6 (uni)<br>Extra : 3.9 ± 0.5 (uni)	– / –	Intra: 13.1 ± 10.4 months<br>Extra: 21 ± 14.7 months	Intra: 100%<br>Extra: 94.9%	Number of febrile UTIs	Intra: 0.17 ± 0.3<br>Extra: 0.22 ± 0.4<br>(No difference)
**Haid et al., 2016**[9]	101 (total)/ 53 Cohen	Cohen: 34.3 months	Multi; C; Retro (matched for VUR grades)	Intra: Open Cohen<br>Extra: Open Mathisen	Total: I-V (uni+ bi)<br>Cohen: 66.7% ≥ III	Yes / –	Intra: 28.2 months	Intra: 98.2%	Febrile and non-febrile UTIs	Intra: 19.2%<br>(5.6% only febrile UTIs)
**Esposito et al., 2016**[10]	90 (total)/ 30 Cohen	Total: 4.86 ± 2.6 years	Multi (2 centers); C; Retro	Intra: Open Cohen<br>Extra: LAP LG<br>Endoscopic STING	Total: II-V (uni+ bi)<br>Cohen: III-V (uni+ bi)	Yes / Yes (CAP if UTI present)	Intra: 3.1 years<br>Range: 1–5 years	Intra: 83.4% (at 6 months)	UTI: not clearly defined	Intra: 6.66%
**Sriram & Babu, 2016**[11]	118 (total)/ 67 Cohen + 51 Modified LG	Cohen: 36 months<br>Modified LG: 15 months	Mono; C; Retro	Intra: Open Cohen<br>Extra: Modified LG	Total: III-V (uni+ bi)<br>Cohen (bi)	Yes / –	Mean: 3 years<br>Range: 1–7 years	Intra: 92.5% (at 3 months)<br>Extra: 92.15% (at 3 months)	Febrile UTIs	Intra: 0%<br>Extra: 0%
**Dogan et al., 2014**[12]	398 (total)/ 373 Intra (367 Cohen) + 25 LG	Total: 59.2 ± 39.8 months	Mono; C; Retro	Intra: Open Cohen + Glenn-Anderson<br>Extra: Open LG	Total: I-V (uni+ bi)	Yes (prophylactic) / Yes (if UTI present)	Total: 25.6 ± 23.3 months	Intra**: 92.7%<br>Extra**: 91.9%	Any kind of UTI and/or symptoms	Intra: 26.7%<br>Extra : 37.5%
**Garcia-Aparicio et al., 2013**[13]	41 (total)/ 19 Cohen	Cohen: 58.79 (41.8-75.8) months	Mono; C; RCT	Intra: Open Cohen<br>Endoscopic injection	Total: II-IV (uni+ bi)	Yes / Yes (prophylactic until resolution on VCUG)	Range: 1–5 years	Open Cohen: 100%	Febrile UTIs	Intra: 0%
**Smith et al., 2011**[14]	50 (total)/ 25 Cohen	Cohen: 69 (3-144 months)	Mono; C; Retro (matched by age and weight)	Intra: Open Cohen<br>Extra: RALUR LG	Total: I-V (uni+ bi)	Yes (in some cases) / Yes (prophylactic until VUR resolution or 3 months)	Mean: 29 months<br>Range: 6–41 months	Intra open Cohen: 100.0%	Febrile UTIs	Intra: 0%
**Marchini et al., 2011**[15]	78 (total)/ 22 Intra + 17 Extra	Intra: 8.8 ± 4.8 years<br>Extra: 6.1 ± 2.7 years	Mono; C; Retro (case-matched study)	Intra: Open<br>Extra: Open<br>Intra: RALUR Glenn-Anderson or Cohen<br>Extra: RALUR LG	Total: II-V (uni+ bi)	Yes / –	Range: 3–24 months	Intra open: 93.2%<br>Extra open: 94.2%	Febrile and non-febrile UTIs	Intra open: 9%<br>Extra open: 0%
**Oberson et al., 2007**[16]	130 (total)/ 74 Cohen	Total: 2.6 years (2 months - 11.8 years)	Mono; C; Retro	Intra: Open Cohen<br>Endoscopic injection	Total: I-V (uni+ bi)	Yes (in some cases) / Yes (10-day CAP, then prophylaxis until VCUG resolution at 6-month)	Mean: 50 months<br>Range:12–104 months	Open Cohen: 96% (at 6 months)	Febrile UTIs***	Intra: 23%
**Nelson et al., 2013**[17]	1076 (total)	5.06 ± 3.61	Mono; NC; Retro	Ureteral reimplantation (Intra+Extra)	Total: I-V (uni+ bi)	Yes / Yes	Median = 21 months	93.5%	Febrile and non-febrile UTIs	21.8% (6.3% only febrile UTIs)

Dash indicates that data were not reported in the article.

* positive urine culture (>10.5 cfu/mL), leukocytes (>10.4/mL), fever (>38.5 °C), CRP, C-reactive protein (>50 mg/L) ; ** Clinical improvement (Disappearance of reflux on VCUG or absence of UTI/symptoms without VCUG); *** Bacteriuria in lower UTI ($\geq 10^5$ bacteria/ml with fever <38.5 °C), pyelonephritis in upper UTI ($\geq 10^5$ bacteria/ml, fever >38.5 °C) and bacterial monoculture; ATB: antibiotic; VUR: vesicoureteral reflux; UTI: urinary tract infection; Intra: intravesical technique; Extra: extravesical technique; Multi: multicentric; Mono: monocentric; C: comparative; NC: Non-comparative; Retro: retrospective; RCT: Randomized controlled trial; LG: Lich-Gregoir; LAP: Laparoscopic; STING: Subureteric Teflon injection; RALUR: Robot-assisted laparoscopic ureteral reimplantation; uni : Unilateral ; bi : Bilateral; CAP: Continuous antibiotic prophylaxis; VCUG: Voiding Cystourethrogram

Table S5 (Continued)

Author, y	N	Study techniques	Preop VUR grade or mean ± SD (laterality)	Inclusion/exclusion criteria	Surgery indication
**Tessier et al., 2022[6]**	171 (total)/77 Intra	Intra: Open Cohen<br>Extra: LAP LG<br>Endoscopic injection	Total: III (uni+ bi)	**Inclusion:** Children (0–18 years) with grade III VUR, febrile UTIs despite ATB or DMSA impact<br>**Exclusion:** Neurogenic bladder, duplex system, PUV, bladder exstrophy	Febrile UTI
**Aydin et al., 2020[7]**	38 (total)/ 18 Intra + 20 Extra	Intra: Cohen<br>Extra: LG	Total: III-V (uni)<br>Intra: 3.7 ± 0.8 (uni)<br>Extra: 3.9 ± 0.8 (uni)	**Inclusion:** Primary unilateral VUR, open UNC (2012–2018), no megaureter<br>**Exclusion:** Tapering, repeat procedures, megaureter, neurogenic bladder	Recurrent VUR post-injection, symptomatic high-grade reflux, antibiotic complications, renal scarring, reflux nephropathy, parental preference
**Silay et al., 2018[8]**	58 (total)/ 23 Intra + 35 Extra	Intra: Cohen<br>Extra: LG	Total: III-V (uni)<br>Intra: 3.4 ± 0.6 (uni)<br>Extra : 3.9 ± 0.5 (uni)	**Inclusion:** Primary unilateral VUR<br>**Exclusion:** Neurogenic bladder, megaureter, tapering, re-operations	Symptomatic high-grade reflux refractory to treatment; recurrent VUR post-injection
**Haid et al., 2016[9]**	101 (total)/ 53 Cohen	Intra: Open Cohen<br>Extra: Open Mathisen	Total: I-V (uni+ bi)<br>Cohen: 66.7% ≥ III	**Inclusion:** Primary VUR<br>**Exclusion:** Prior antireflux treatment, neurogenic bladder, non-refluxive megaureters	BUTIs, persistence of VUR, post pyelonephritic changes in DMSA
**Esposito et al., 2016[10]**	90 (total)/ 30 Cohen	Intra: Open Cohen<br>Extra: LAP LG<br>Endoscopic STING	Total: II-V (uni+ bi)<br>Cohen: III-V (uni+ bi)	**Inclusion:** Grade II–V primary VUR<br>**Exclusion:** Grade V with megaureter, prior VUR surgery, significant anomalies, tapering	Recurrent febrile UTI: BUTIs despite CAP or after CAP interruption, renal scarring or 20% decreased function (vs contralateral kidney) on DMSA
**Sriram & Babu, 2016[11]**	118 (total)/ 67 Cohen + 51 Modified LG	Intra: Open Cohen<br>Extra: Modified LG	Total: III-V (uni+ bi)<br>Cohen (bi)	**Inclusion:** Children with VUR reimplantation (2003–2014).<br>**Exclusion:** Secondary VUR, duplication, ectopic ureter, or no follow-up	Recurrent BUTIs, worsening scars
**Dogan et al., 2014[12]**	398 (total)/ 373 Intra (367 Cohen) + 25 LG	Intra: Open Cohen + Glenn-Anderson<br>Extra: Open LG	Total: I-V (uni+ bi)	**Inclusion:** VUR reimplantation (2001–2012)<br>**Exclusion:** Prior open surgery, secondary VUR	Recurrent febrile UTI on prophylaxis, unlikely to resolve spontaneously, 70.7% preoperative renal scarring, prior endoscopic injection, parental preference
**Garcia-Aparicio et al., 2013[13]**	41 (total)/ 19 Cohen	Intra: Open Cohen<br>Endoscopic injection	Total: II-IV (uni+ bi)	**Inclusion:** Primary VUR, aged over 1 year<br>**Exclusion:** ESRD, poor renal function (<15% DRF), solitary kidney, complex uropathy, neurogenic bladder	Recurrent UTI, persistent VUR after 2 years of prophylaxis, impaired renal function
**Smith et al., 2011[14]**	50 (total)/ 25 Cohen	Intra: Open Cohen<br>Extra: RALUR LG	Total: I-V (uni+ bi)	**Inclusion:** VUR patients with RALUR or open UR (2006–2009)<br>**Exclusion:** Ectopic ureters	BUTIs, persistence of VUR, progressive renal scarring, parental preference
**Marchini et al., 2011[15]**	78 (total)/ 22 Intra + 17 Extra	Intra: Open<br>Extra: Open<br>Intra: RALUR Glenn-Anderson or Cohen<br>Extra: RALUR LG	Total: II-V (uni+ bi)	**Inclusion:** Primary VUR grade II–V with breakthrough pyelonephritis or worsening renal function/scars despite prophylaxis, Hutch diverticulum<br>**Exclusion:** Obstructed megaureter, ureterocoele, PUV, neurogenic bladder	Breakthrough pyelonephritis, worsening renal function, or scars despite prophylaxis
**Oberson et al., 2007[16]**	130 (total)/ 74 Cohen	Intra: Open Cohen<br>Endoscopic injection	Total: I-V (uni+ bi)	**Inclusion:** Children treated with endoscopic subureteral collagen injection or Cohen reimplantation (1992–1999)	Intra: BUTIs, high-grade reflux, impaired renal function
**Nelson et al., 2013[17]**	1076 (total)	Ureteral reimplantation (Intra+Extra)	Total: I-V (uni+ bi)	**Inclusion:** Open ureteral reimplantation for primary VUR (radiographic grade I–V or scintigraphic grade 1–3)<br>**Exclusion:** Secondary VUR, repeat, laparoscopic, or endoscopic procedures	BUTIs, persistent VUR, altered differential function, new scarring, or parental preference/non-compliance

Dash indicates that data were not reported in the article.

VUR: Vesicoureteral reflux; Intra: Intravesical technique; Extra : Extravesical technique; LAP: Laparoscopic; LG: Lich-Gregoir; uni : Unilateral ; bi : Bilateral; UTI: Urinary tract infection; ATB: Antibiotic; DMSA: Dimercaptosuccenic scan; PUV: Posterior urethral valves; UNC: Ureteroneocystostomy; BUTIs: Breakthrough urinary tract infections; STING: Subureteric Teflon injection; CAP: Continuous antibiotic prophylaxis; ESRD: End-stage renal disease; DRF: Differential renal function; RALUR: Robot-assisted laparoscopic ureteral reimplantation; UR: Ureteral reimplantation

Table S6

Age and follow-up duration comparison by sex.

	Male	Female	p-value
**Distribution, n (%)**	230 (56.9)	174 (43.1)	**0.004**[a]
**Median age (range), years**	7 (1-17)	8 (1-18)	0.067[b]
**Mean age (SD), years**	7.04 (2.92)	7.53 (2.42)	0.067[b]
**Median follow-up (range), years**	2.5 (0.9-10.9)	2.2 (0.9-11.2)	**0.002**[b]
**Mean follow-up (SD), years**	3.7 (3.01)	2.9 (2.3)	**0.002**[b]

[a]Using a chi-square goodness-of-fit test; [b]Using Student's t-tests
